# Appreciation of Time and Emotional Well-Being in Adulthood

**DOI:** 10.1093/geroni/igaf122.346

**Published:** 2025-12-31

**Authors:** Claire Growney, Tyler Matteson, Laura Carstensen

**Affiliations:** Stanford University, Stanford, California, United States; Stanford University, Stanford, California, United States; Stanford University, Stanford, California, United States

## Abstract

According to socioemotional selectivity theory, as individuals age and experience time as increasingly limited, appreciation for remaining time in life increases. Theoretically, this leads people to prioritize meaningful experiences and social relationships that contribute to emotional well-being. To examine this postulate, we developed the Appreciation of Remaining Time (ART) scale and established its convergent and discriminant validity. The present study examined the relationship of the ART scale with existing measures of savoring and measures of well-being. 406 participants aged 18–90 (Mage = 49.33, SD = 19.28) completed the ART scale, as well as measures of time perspective, savoring, and gratitude. Participants also completed assessments of emotional well-being and a social preference paradigm task. Analyses established discriminant validity for the ART scale from measures of time perspective, savoring, and gratitude. ART mediated associations between age and outcomes of interest. Specifically, age was associated with greater appreciation of remaining time, which in turn was associated with greater emotional well-being and stronger preferences for engaging with emotionally meaningful social partners. These findings replicate and extend prior research, suggesting that the ART scale taps a distinct construct that holds meaningful implications for socioemotional aging. Viewing time as both limited and valuable appears to contribute to greater emotional well-being at older ages.

